# A low‐cost multimodal head‐mounted display system for neuroendoscopic surgery

**DOI:** 10.1002/brb3.891

**Published:** 2017-12-14

**Authors:** Xinghua Xu, Yi Zheng, Shujing Yao, Guochen Sun, Bainan Xu, Xiaolei Chen

**Affiliations:** ^1^ Department of Neurosurgery Chinese PLA General Hospital Beijing China; ^2^ Department of Dermatology Beijing Chaoyang Hospital Capital Medical University Beijing China

**Keywords:** head‐mounted display, neuroendoscopic surgery, surgical assistant instruments, virtual endoscopy

## Abstract

**Background:**

With rapid advances in technology, wearable devices as head‐mounted display (HMD) have been adopted for various uses in medical science, ranging from simply aiding in fitness to assisting surgery. We aimed to investigate the feasibility and practicability of a low‐cost multimodal HMD system in neuroendoscopic surgery.

**Methods:**

A multimodal HMD system, mainly consisted of a HMD with two built‐in displays, an action camera, and a laptop computer displaying reconstructed medical images, was developed to assist neuroendoscopic surgery. With this intensively integrated system, the neurosurgeon could freely switch between endoscopic image, three‐dimensional (3D) reconstructed virtual endoscopy images, and surrounding environment images. Using a leap motion controller, the neurosurgeon could adjust or rotate the 3D virtual endoscopic images at a distance to better understand the positional relation between lesions and normal tissues at will.

**Results:**

A total of 21 consecutive patients with ventricular system diseases underwent neuroendoscopic surgery with the aid of this system. All operations were accomplished successfully, and no system‐related complications occurred. The HMD was comfortable to wear and easy to operate. Screen resolution of the HMD was high enough for the neurosurgeon to operate carefully. With the system, the neurosurgeon might get a better comprehension on lesions by freely switching among images of different modalities. The system had a steep learning curve, which meant a quick increment of skill with it. Compared with commercially available surgical assistant instruments, this system was relatively low‐cost.

**Conclusions:**

The multimodal HMD system is feasible, practical, helpful, and relatively cost efficient in neuroendoscopic surgery.

## 1 INTRODUCTION

Neuroendoscopic surgery becomes widely accepted with the publication of results in large clinical series in the late 1990s and has been an expanding field of neurosurgery (Cinalli et al., 1999; Hidalgo, Ali, Weiner, & Harter, 2016; Hopf, Grunert, Fries, Resch, & Perneczky, 1999). Neuroendoscopic surgery reflects the tendency of modern neurosurgery toward minimum injury, which represents access and visualization through the narrowest practical corridor and maximum effective action at the target area with minimal disruption of normal tissues (Elbabaa, 2016). With the advantages of a wider vision of surgical field, less traumatism to normal brain tissues, and reduced complications, neuroendoscopic surgery is gaining more and more popularity in neurosurgeons and patients. It has become the first choice of management for pituitary adenoma and intraventricular pathologies such as obstructive hydrocephalus, intracranial cysts, and small intraventricular tumors (Di Rocco, Yoshino, & Oi, 2005; Rocque, 2016).

However, everything has its limitations. For one thing, neuroendoscope generally offers two‐dimensional (2D) image and has no depth of field compared with microscope. For another, the learning curve of neuroendoscopic surgery is long and shallow, which means a longer learning time (Bokhari, Davies, & Diamond, 2013). Furthermore, the monitor of a neuroendoscope is usually connected together with xenon light source and digital video recording system, which is large and ponderous, making it inconvenient to adjust the location freely during operation. Using a standard monitor sometimes requires surgeons to move into unpleasant postures.

In neurosurgery, it is of key importance to have a thorough, accurate, and detailed knowledge of the anatomical structure of the surgical target because the surgical outcomes are directly connected to patient's neurological functions. Compared with 2D methods, 3D computer simulation enables more accurate, realistic, and intuitive diagnosis and surgical analysis (Khor et al., 2016). With rapid advances in technology, virtual reality, augmented reality, and wearable devices have been adopted for various uses in medicine, ranging from simply facilitating fitness to more complex devices used in surgery (Liu, Jenkins, Sanderson, Fabian, & Russell, 2010; Maithel, Villegas, Stylopoulos, Dawson, & Jones, 2005; Shao et al., 2014). In medicine, wearables can be broadly divided into body sensors and head‐mounted displays (HMD). During operative procedures, HMDs can provide surgeons with 3D radiographic information while maintaining sterility.

To overcome the deficiencies mentioned above, we developed a low‐cost, easy to use multimodal HMD system on the strength of wearable devices and computer image processing technology. Feasibility and practicability of such a system were investigated in neuroendoscopic surgeries involving ventricular system diseases. A detailed introduction of the system was made in this study.

## 2 MATERIALS AND METHODS

### 2.1 Constitution of the multimodal HMD system

**Figure 1:**
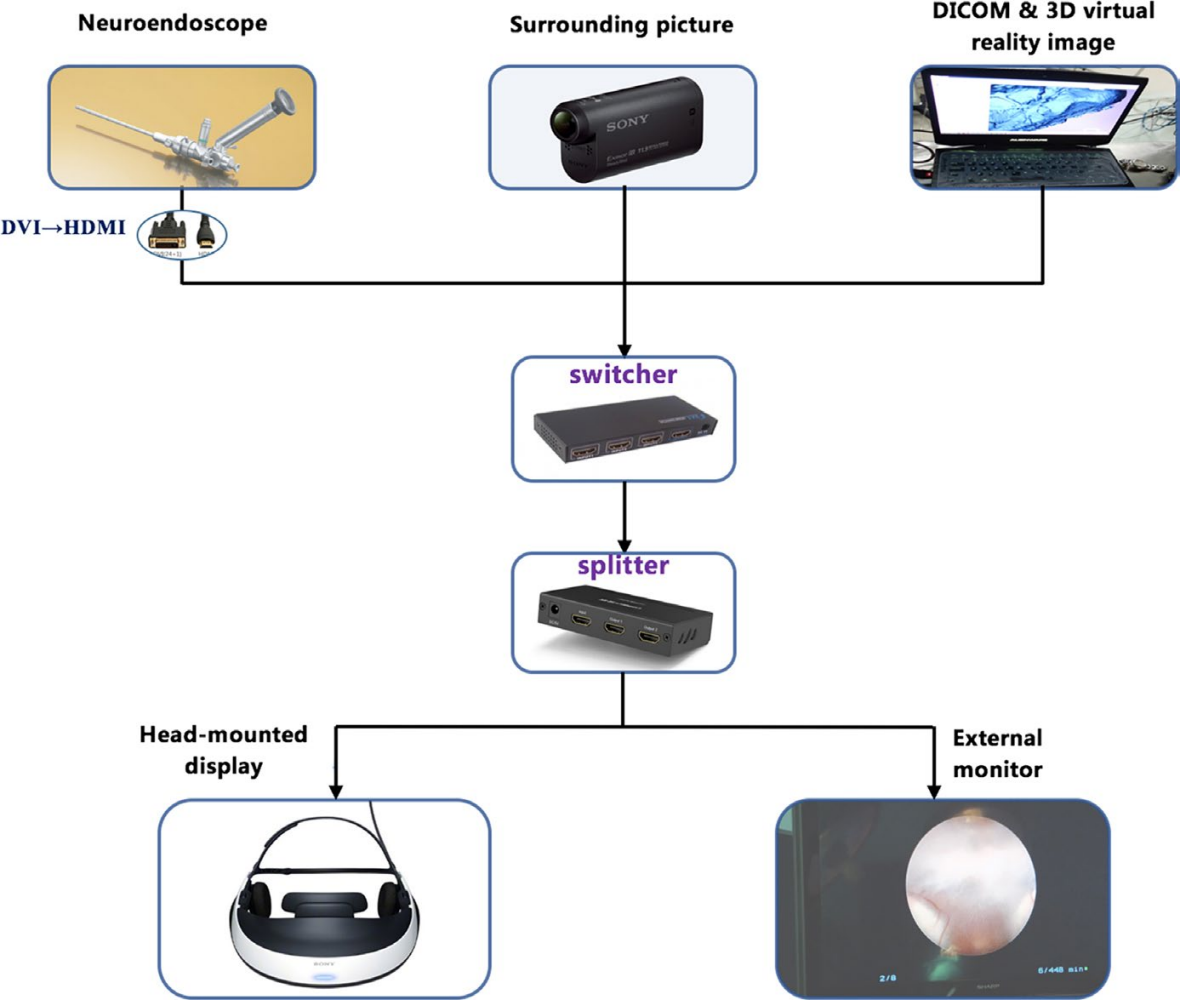
Schematic view of the overall configuration of the multimodal head‐mounted display system for neuroendoscopic surgery. DVI, digital video interactive; DICOM, digital imaging and communications in medicine

The system mainly contained three components: a HMD, an action camera, and a laptop computer, all of which were commendably connected together. Concrete connection pattern and detailed specification information of instruments were presented in Figure 1 and Table 1. The HMD, a HMZ‐T1 Personal 3D Viewer (Sony, Japan) to be specific, was the critical part of this system. HMZ‐T1 is a visor style HMD composed of a visor and an external processor unit. It contained two miniature organic light emitting displays (size: 0.7 inch, resolution: 1,280 × 720px per eye) providing video. It could display any proprietary video compatible with the high‐definition multimedia interface (HDMI) standard. By connection with the neuroendoscope, the neurosurgeon could get a high‐definition or 3D image of surgical field, getting rid of the restriction of monitor position. By combining with computer image processing technique, we could realize 3D display and virtual endoscopy (VE) simulation of ventricular diseases.

**Table 1:** Hardware components of the multimodal head‐mounted display system

Components	Description
HMD	Sony HMZ‐T1, display resolution: 1,280 × 720 pixels per eye
Action camera	Sony HDR‐AS20, 170° ultrawide lens, and 1,080p video recording
Laptop computer	Lenovo 310, Intel Core i5 2.7 GHz, 8 GB SDRAM, and 500 GB HDD
Leap motion controller	Leap motion, submillimeter hand tracking, 150° field of view, and 3D interaction
Switcher	Ugreen, 1,080p, three HDMI input ports, one HDMI output port, and infrared radiation remote control
Splitter	Ugreen, 1,080p, one HDMI input port, and two HDMI output ports

1,080p, 1,920 × 1,080 pixels; HDD, hard disk drive; HDMI, high‐definition multimedia interface; SDRAM, synchronous dynamic random access memory.

To realize real‐time visualization of surrounding environment on the premise not taking off the HMD repeatedly, an action camera (HDR‐AS20, Sony, Japan) was bounded to the HMZ‐T1 viewer. With a 170° ultrawide lens, the HDR‐AS20 could record video of 1,080p with intense contrast and sharpness. Fastened to side arm of HMZ‐T1, the action camera was like a pair of eyes observing the surrounding environment. With the action camera, the neurosurgeon could perceive the surroundings whenever needed by just switching the input sources rather than putting on and taking off the HMD repeatedly.

The HMZ‐T1 processor was a small set‐top box with only one input and one output. But there were three different image sources (the endoscope image, the 3D VE image, and real‐time video of surroundings) to display. To overcome this defect, both HDMI switcher (Ugreen, China) and HDMI splitter (Ugreen, China) were used at the same time. Through the switcher, neuroendoscope, laptop computer, and action camera were connected to the HMD all together. With the splitter, collected images were displayed on both the HMD and the monitor of neuroendoscope simultaneously so that the assistants and nurses could get the same view with the operator. Combining all these instruments together, the neurosurgeon could freely switch among different image sources using a sterilized remote controller of the switcher without moving cables around.

### 2.2 Acquisition and processing of radiological images

**Figure 2:**
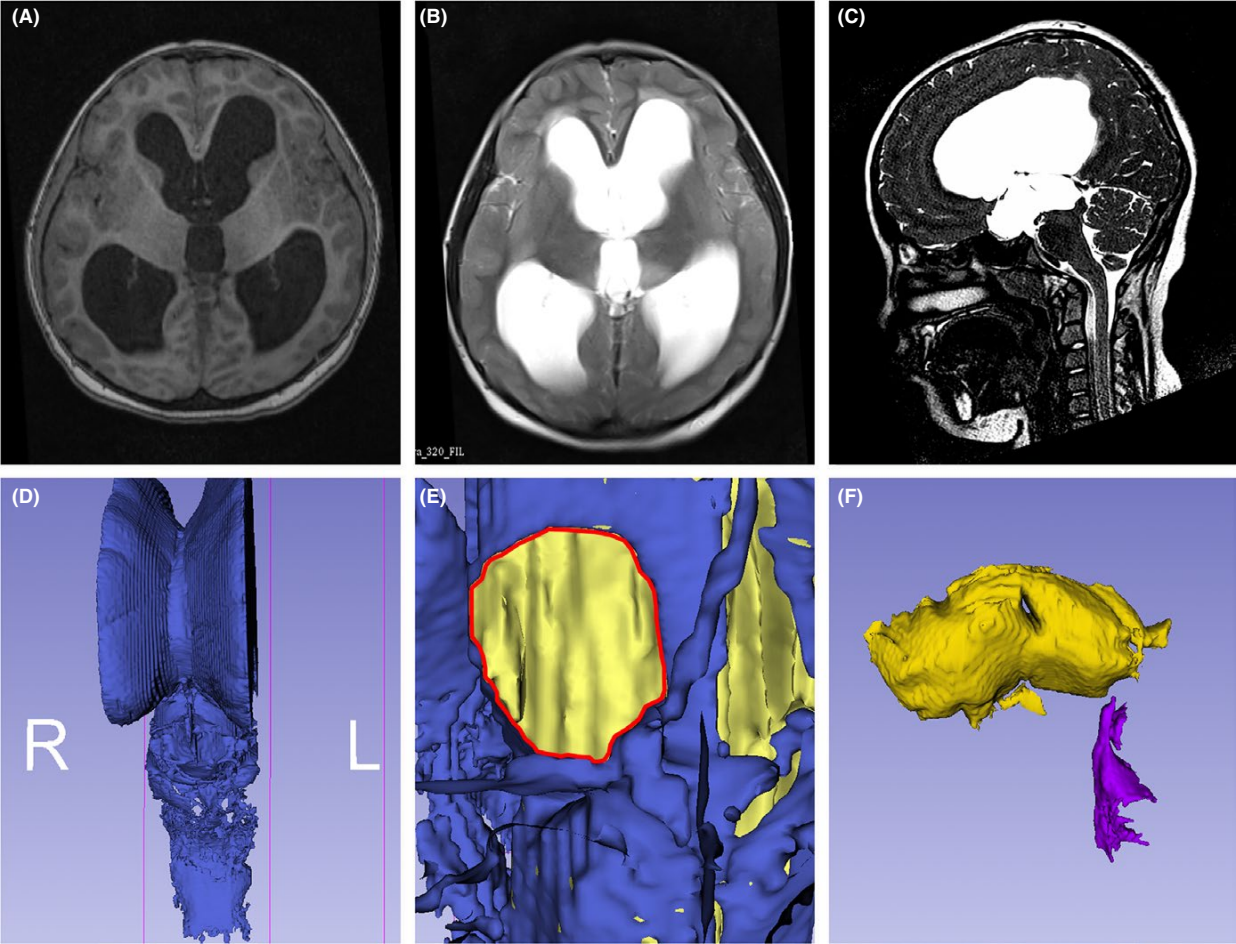
Patient's MRI image and reconstructed virtual endoscopy image of cerebral ventricles. (A and B) T1 and T2 images showed lateral ventricles, and the third ventricle had enlarged, and the surrounding brain tissue was oedematous; (C) 3D‐SPACE image showed that the cerebral aqueduct seemed obstructed; (D) 3D reconstruction of ventricular system with software 3D Slicer; (E) virtual endoscopy image demonstrated that the interventricular foramen (red line) was enlarged; (F) the third ventricle failed to connect to the fourth ventricle because the cerebral aqueduct was obstructed

All image data processing was performed with 3D Slicer (https://www.slicer.org, version 4.5.0), an open source software platform for medical image informatics, image processing, and 3D visualization. To acquire appropriate MRI data for processing, all patients routinely underwent both conventional and 3D sampling perfection with application optimized contrast (3D‐SPACE) using different flip angle evolution MRI examination performed on the same 1.5‐T MRI scanner (Siemens Espree, Germany) (Li et al., 2016). The imaging protocol started with a T1‐weighted, 3D magnetization prepared rapid acquisition gradient echo (MPRAGE) sequence (repetition time [TR]/echo time [TE], 1,650/3 ms; slice thickness, 1 mm; field of view [FOV], 250 mm; matrix size, 256 × 256), followed by transverse and sagittal T2‐weighted (TR/TE, 6,000/95 ms; slice thickness, 5 mm; FOV, 230 mm; matrix size, 256 × 160). For cyst detection and VE reconstruction, 3D‐SPACE imaging (TR/TE, 1,500/226 ms; slice thickness, 0.74 mm; FOV, 225 mm; flip angle, 130°) was used in addition to high‐resolution heavy T2‐weighted images. The preoperative imaging data in Digital Imaging and Communications in Medicine format were then transferred to a laptop computer (Intel Core i5, 2.7 GHz; RAM: 8 GB; Lenovo, China) to reconstruct VE images using software 3D Slicer. The reconstructed VE images might help the neurosurgeon to better define location and characteristics of lesions intuitively (Figure 2).

### 2.3 Applications in neuroendoscopic surgery

**Figure 3:**
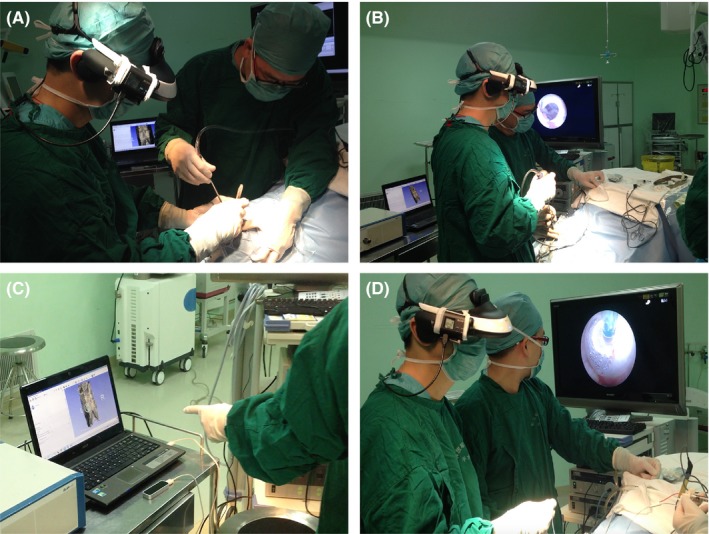
Endoscopic third ventriculostomy with the help of head‐mounted multimodal display system. (A) Keyhole craniotomy under action camera view; (B) endoscope‐assisted observation of intraventricular structure; (C) observation and adjustment of patient's reconstructed 3D virtual endoscopy image; (D) creation of an opening in the floor of the third ventricle under endoscopic view

It took just several minutes to assemble the multimodal HMD system in a surgery. The neurosurgeon first employed the action camera view to accomplish surgical procedures that did not need the neuroendoscope, such as scalp incision and trepanning. Once the neuroendoscope was placed into the ventricle, endoscopic image would be displayed on the HMD. When the neurosurgeon wanted to look through patient's preoperative MRI image or reconstructed 3D VE image to better understand the positional relation between lesions and surrounding normal tissues, all he needed to do was to switch the input source to laptop computer. Details on how the system worked in a neuroendoscopic surgery are shown in Figure 3.

The study was approved by the Institutional Review Board of Chines PLA General Hospital and was conducted in conformity to the Declaration of Helsinki. All patients gave their written informed consent prior to participation in this study.

### 2.4 Statistical analysis

The quadratic regression analysis of operative time and number of cases performed was used to determine the learning curve for a neurosurgeon to become adept at multimodal HMD system assisted neuroendoscopic surgery. Statistical analyses were performed using SPSS 22.0 software (SPSS, USA).

## 3 RESULTS

### 3.1 Demographic and clinical characteristics

Between March 2015 and December 2016, a total of 21 consecutive patients with ventricular diseases underwent neuroendoscopic surgeries with the help of this multimodal HMD system in our department. Their average age was 20.1 years (range, 1–53 years). The disease category contained obstructive hydrocephalus (eight cases), suprasellar arachnoid cyst (seven cases), pineal region tumors (five cases), and Dandy‐Walker malformation (one case). Interestingly, five patients with suprasellar arachnoid cyst had been previously misdiagnosed with other hydrocephalus‐causing diseases, and four had been previously treated with ventriculoperitoneal shunt with no symptom relief. All MRI scans and medical image processing were performed prior to surgery. It took 12 min on average to reconstruct a 3D VE image of one patient while assembly of the system cost 5–10 min.

Demographic and clinical characteristics of included patients are summarized in Table 2. Neuroendoscopic surgery was safely and successfully implemented in all cases and system‐related complications occurred in no patients. After surgery, one patient suffered from transient mild oculomotor paralysis, which completely recovered 2 weeks later. None of the patients had intracranial infection. Clinical improvement was observed in all patients after surgery. After an average follow‐up of 14.7 months (range, 5–30 months), no patients needed a reoperation.

**Table 2:** Demographic and clinical characteristics of included patients

Case no.	Gender	Age, years	Main symptoms	Diagnosis	Operation method	Clinical improvement	Follow‐up, months
1	F	15	Headache	OH	ETV	Yes	9
2	M	43	Headache	PRT	ETV + tumor biopsy	Yes	18
3	F	18	Seizure	SAC	VCC	Yes	30
4	M	18	Visual defect	SAC	VCC	Yes	14
5	M	1	Developmental delay	OH	ETV	Yes	10
6	F	47	Headache	PRT	ETV + tumor biopsy	Yes	11
7	F	24	Seizure	OH	ETV	Yes	12
8	M	23	Headache	OH	ETV	Yes	24
9	M	14	Seizure	PRT	ETV + tumor biopsy	Yes	15
10	M	7	Precocious puberty	SAC	VCC	Yes	18
11	M	10	Dizziness	SAC	VCC	Yes	18
12	M	53	Headache	PRT	ETV + tumor biopsy	Yes	16
13	F	42	Visual defect	OH	ETV	Yes	9
14	F	12	Seizure	OH	ETV	Yes	12
15	M	30	Headache	OH	ETV	Yes	27
16	M	15	Headache	PRT	ETV + tumor biopsy	Yes	18
17	F	18	Dizziness	SAC	VCC	Yes	9
18	M	2	Developmental delay	Dandy‐Walker	ETV	Yes	21
19	F	18	Headache	SAC	VCC	Yes	6
20	M	1	Macrocrania, vomiting	SAC	VCC	Yes	6
21	F	11	Dizziness	OH	ETV	Yes	5

ETV, endoscopic third ventriculostomy; F, female; M, male; OH, obstructive hydrocephalus; PRT, pineal region tumor; SAC, suprasellar arachnoid cyst; VCC, ventriculocystocisternostomy.

### 3.2 Feasibility and comfort

Neuroendoscopic surgery was successfully performed under the assistance of the multimodal HMD display system in all patients, with no system‐related technical problem happened. The neurosurgeon (Chen, X. L.) experienced this multimodal HMD as a helpful, comfortable, lightweighted, and easily adjustable device. Switching the display to the action camera modality, the neurosurgeon could perform surgical procedures and make contact with the environment easily as usual. When switched to endoscope modality, the endoscopic image was always in the direct line of sight, allowing the neurosurgeon to work in a relaxed position. When the neurosurgeon would like to look over patient's medical images, all he needed is to switch the input source to computer using a sterilized remote controller.

Eye fatigue occurred in three patients whose operation time exceeded 80 min at the early stage of this study. No complaints of blurred vision, dizziness, or other forms of simulation sickness arose. During operation, the neurosurgeon could adjust or rotate the reconstructed 3D VE image whenever necessary with the assistance of a leap motion controller connected to the computer (Figure 3C). The VE view during operation helped the surgeon to get a better understanding of spatial relationships between lesions and surrounding anatomic structures. The ability of freely switching among endoscope images, VE images, and surrounding images was a key feature of this system.

### 3.3 Displays and learning curve

**Figure 4:**
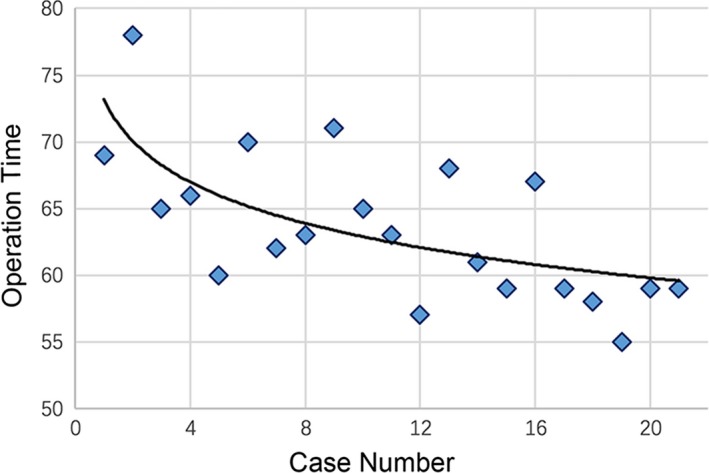
Learning curve of the multimodal head‐mounted display system. Quadratic regression analysis of the operative time and number of cases experienced

Screen resolution of the HMD was high enough for the neurosurgeon to judge lesions and peripheral structures and to operate surgical equipment accurately. The range and depth of vision of the system seemed comparable to or even better than those of the video monitor. Quadratic regression analysis of operation time and the number of cases showed a steep learning curve (Figure 4), which meant that the system could be learnt and mastered in a short time. Operation time decreased sharply to a stable level with the increase of operations performed, which meant that the system was easy to master. The multimodal HMD system was feasible and easy to use with sufficient safety for neuroendoscopic application.

## 4 DISCUSSION

This study proposed an innovative wearable multimodal display system for easy switching between endoscopic, surrounding, and computerized image modalities in neuroendoscopic surgery. Wearable technology is an emerging industry which has made possible the integration of technology into healthcare and surgery as well as daily life. The trend has been supported by the miniaturization of the components necessary for the collection, storage, processing, sharing, and presentation of data (Ortega, Wolff, Baumgaertner, & Kendoff, 2008; Rawassizadeh et al., 2015). Several HMD devices have been identified, which are currently utilized in surgery, imaging, simulation, education, and as a navigation tool (Banaee, Ahmed, & Loutifi, 2013; Inoue et al., 2015; Kim, Kim, & Kim, 2017; Maithel, Villegas, Stylopoulos, Dawson, & Jones, 2005; van Koesveld, Tetteroo, & de Graaf, 2003). Wearable technology, properly applied, could provide a more objective glimpse into the true nature of a patient's illness, allowing improvements in shared decision‐making (Iqbal, Aydin, Brunckhorst, Dasgupta, & Ahmed, 2016).

### 4.1 Multimodality medical image

Multimodality medical image fusion and processing have developed rapidly in recent years. Multimodality medical image and postprocessing have proved to be clinically useful for accurate diagnosis and effective surgical treatment of brain disease (Giordano, Wrede, Stieglitz, Samii, & Lüdemann, 2007; Ito et al., 2010). Reconstructed images can be adjusted and rotated freely, not only providing a better representation of spatial relationships between lesions and surrounding structures but also predicting the patterns and variants that will be encountered on the trajectory to lesions (Kakizawa et al., 2003). Moreover, reconstructed images allow the surgeon to depict the actual operative window from angles imitating surgical approaches and to understand the neurovascular complex by viewing from angles that are impossible to obtain intraoperatively (Neubauer & Wolfsberger, 2013; Satoh, Onoda, & Date, 2007; Xu et al., 2014). 3D visualization can provide the surgeon with individualized, objective anatomic information. In this study, the multimodal HMD system realized free switching between different modal images. The reconstructed VE images provide subtle visual cues such as shadow effects, depth perception, and highlighting results, which allow for an interactive evaluation by zooming, panning, and rotating (Haerle et al., 2015; Zhao et al., 2015). By comparing VE image with actual endoscope image, the neurosurgeon can get a better comprehension of the positional relation between lesions and normal brain tissues, enabling more accurate, realistic, and intuitive surgical analysis.

### 4.2 Working condition in surgery

Working condition in surgery should enable the surgeon to perform an operation under relaxed position with minimal muscular strain (Cutolo et al., 2017; Hanna, Shimi, & Cuschieri, 1998). In our opinion, the use of such a multimodal display system seemed superior to standard monitor in neuroendoscopic surgery. The orientation of the head and eyes had a natural inclination toward gaze‐down viewing in the direction of the hand. Wearing the HMD, the neurosurgeon could stand in his/her favorable position. When input source was switched to surrounding image, what the action camera recorded was adequate enough for the neurosurgeon to operate the instruments and communicate with assistants or nurses. There was no more need to constantly move the screen out of the way and plug it back later on. It was an advantage that with this system the monitor no longer needed to be moved repeatedly to find a better view for the surgeon when his/her position is changed.

Comfort and expenditure of the system were also important factors to be considered. The HMZ‐T1 was not very heavy (14.8 ounces) and accommodated glasses very well. To our experience, it was pleasant to wear that weight on the head for 1.5 hr or a shorter time, after that, it might make the host discomfort or annoyed. The weight of HMD required improvement. Luckily, most ventricular neuroendoscopic surgeries could be finished within 2 hr. Compared with most commercially available surgical assistant systems, the cost of our system is much less (total cost, exclusive of the computer, approximately $1,200). Moreover, the system had a steep learning curve, which meant that it was easy and quick to learn and master such a multimodal HMD system.

### 4.3 Limitations

There are some limitations that should be acknowledged. The application of wearable devices in neurosurgery is relatively new, and the true usefulness of such a multimodal HMD system needs to be better validated and assessed with prospective controlled study.

## 5 CONCLUSIONS

In this study, we present a low‐cost multimodal HMD system for neuroendoscopic surgery. As shown by the study, the system is feasible, practical, helpful, and relatively cost efficient. Through free switching among different image modalities, the system enables a more relaxed position, a better comprehension of lesion's 3D positional relations, and a better view of the operative field. With advances in technology and instruments, wearable technology would play a more significant role in neuroendoscopic surgery and other surgeries in the near future.

## CONFLICT OF INTEREST

The authors declare that there is no conflict of interest.
